# Genomic characterization of Enterotoxigenic *Escherichia coli* lineage 2 (CS2 + CS3) by long-read sequencing reveals distinct lineage-specific genome organization

**DOI:** 10.1038/s41598-026-55068-w

**Published:** 2026-05-26

**Authors:** Nayyer Taheri, Åsa Sjöling

**Affiliations:** 1 Department of Chemistry and Molecular Biology, University of Gothenburg, Gothenburg, Sweden; 2 Department of Microbiology, Tumor and Cell Biology, Karolinska Institute, Stockholm, Sweden

**Keywords:** ETEC, L2, CS2, CS3, ARGs, Genetics, Microbiology

## Abstract

Enterotoxigenic *Escherichia coli* (ETEC) is a major cause of diarrhoea in children, adults and travellers in endemic regions. ETEC pathogenesis is mediated by heat-labile (LT) and heat-stable (ST) enterotoxins together with colonization factors. In this study, we characterized the chromosomal and plasmid features of twelve ETEC lineage 2 (L2) isolates, including five isolates sequenced by long-read PacBio technology and reported for the first time in this study, and seven publicly available complete genomes. Comparative genomic analysis revealed a highly conserved chromosomal backbone and two core plasmids, with the CS2 operon uniquely integrated into the chromosome, while other virulence determinants, including LT, ST, CS3, EatA, CS21, and EtpBAC remained plasmid-encoded. Fusion and consolidation of lineage 1-like plasmids produced a streamlined plasmidome in L2, preserving essential virulence genes while occasionally incorporating antibiotic resistance determinants. Phage-like plasmids were identified sporadically, highlighting ongoing horizontal gene acquisition. These findings demonstrate that ETEC L2 employs a hybrid virulence architecture combining chromosomal fixation of a key colonization factor with plasmid-mediated accessory traits, potentially enhancing stability and transmissibility. The study provides new insights into the genomic organization, evolutionary dynamics, and pathogenic potential of this emerging ETEC lineage.

**Supplementary Information:**

The online version contains supplementary material available at 10.1038/s41598-026-55068-w.

## Introduction

*Escherichia coli* is a highly diverse bacterial species encompassing both commensal and pathogenic lineages. Among the pathotypes, enterotoxigenic *E. coli* (ETEC) is a significant cause of acute diarrheal disease worldwide, particularly affecting children under five in resource-limited regions and travellers to endemic area^1–3^. ETEC is responsible for sporadic food-borne gastroenteritis outbreaks, causing an estimated 10 million cases annually^4^.

ETEC pathogenicity is primarily associated with plasmid-encoded virulence determinants, including heat-labile (LT) and heat-stable (ST) enterotoxins and a variety of colonization factors (CFs) that mediate adherence to intestinal epithelial surfaces. Plasmid composition and associated virulence determinants are defining features of specific ETEC lineages^5–8^. A majority of ETEC infections are associated with globally disseminated lineages (L1-L7), each defined by specific toxin and colonization factor profiles, including CFA/I and CS1-CS6. These lineages also commonly exhibit conserved serotypes^8^.

Recent studies indicate that ETEC infections remain common and have been increasing since 2022. In England, ETEC infections rose by 35.5% between 2015 and 2023. The most prevalent ETEC pathotypes were isolates belonging to ST182 O169 (L7) and ST4 O6:H16 expressing STp and colonization factor CS6 (L7), and LT/STh and colonization factors CS2 + CS3 (L2), respectively, with both lineages exhibiting multi-drug resistance^9^. A study of ETEC incidence in Zambia reported that LT, ST, CS2 + CS3 and CS6 were the most common virulence types among isolates^10^. Research on travellers’ diarrhoea in adults and US military personnel between 2018 and 2023 found ETEC to be the dominant pathogen in five out of six locations investigated^11^. The same study identified CS3 and CS21, followed by CS2 and CS6, as the most frequent ETEC virulence factors. Collectively, these findings indicate a recent global increase in ETEC isolates positive for CS2 + CS3 and CS6.

Antibiotic resistance in ETEC is increasing globally, with ESBL-producing isolates, particularly those expressing *bla*_CTX−M−15_, emerging over the last decade^12–16^. Multidrug resistance has been frequently reported in CS2 + CS3 L2 isolates, raising public health concerns in regions with high diarrheal disease burden and widespread antibiotic use^9,13^.

Previous studies have shown that ETEC lineages carry large, conserved plasmids encoding virulence factors together with genes involved in antibiotic resistance and stress tolerance^7^. In our previous study, currently under review at *BMC Microbiology*, we characterized the plasmidome of the L1 strains and identified four conserved plasmids contributing to virulence. In contrast, the plasmid content and genomic features of the L2 isolates have remained less well characterized, although preliminary evidence suggests that L2 strains harbour plasmids encoding key virulence factors such as LT, STh, CS3, EatA, EtpBAC, and CS21^7^.

Therefore, the aim of this study was to characterize the chromosomal and plasmid features of L2 isolates to better understand their genomic diversity, pathogenic potential, and contribution to antibiotic resistance dissemination.

## Results

### Chromosomal integration of CS2 revealed lineage-specific rearrangement of colonization factors

**Fig. 1:**
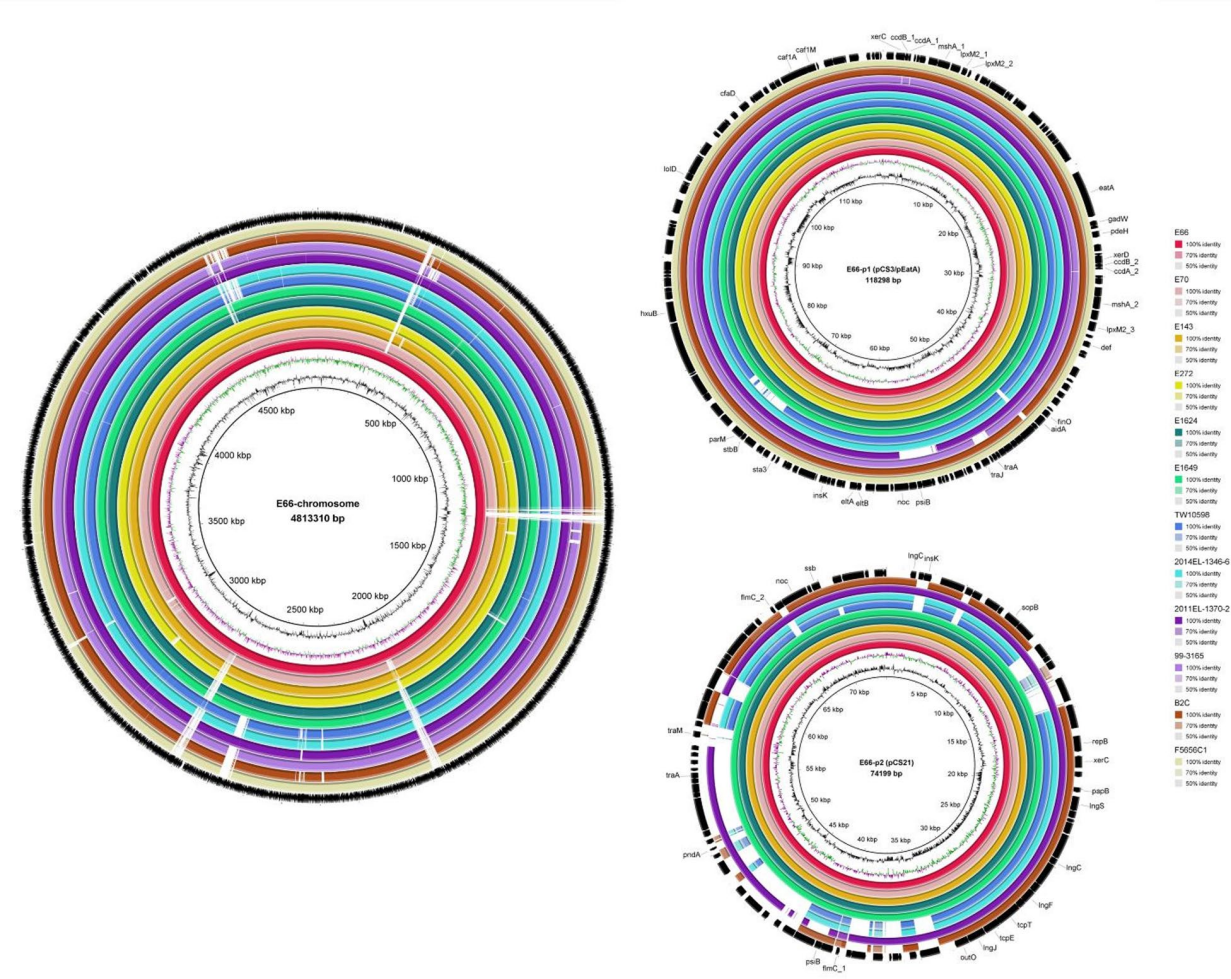
Comparative analysis of chromosome and core plasmids in ETEC L2 isolates. Circular BLASTn alignment of the chromosome (left) and conserved plasmidome (upper right, p1; lower right, p2) from 12 ETEC L2 isolates, using the chromosome and plasmids of strain E66 as reference, visualized with BRIG. The outermost ring represents the annotated E66 chromosome or plasmids, while each concentric inner ring corresponds to an individual L2 isolate aligned against the reference. Ring colour intensity reflects nucleotide identity to E66, with darker shading indicating higher similarity and lighter shading indicating lower similarity; white regions denote sequences absent relative to the reference. The innermost rings display GC content and GC skew of the E66 chromosome.

We examined the genomic architecture of ETEC L2 isolates using a combined dataset of five newly sequenced strains and seven publicly available PacBio-generated genomes (Table 1). Whole-genome sequencing and comparative analysis of the twelve L2 isolates revealed a highly conserved genomic organization. All strains belonged to serotype O6:H16 and possessed a single circular chromosome and two conserved plasmids (Fig. 1, Figure S1). The chromosome was approximately 4.8 Mbp in length, with a GC content of about 51%, and encoded roughly 4,500 protein-coding genes and 22 RNA genes (Table S1).

**Table 1:** Characteristics of the ETEC L2 isolates.

Strain	Sequence type	Toxin profile	CF profile	Collection date	Collection location	Source of data*	Reference
E66	ST-4	LT + STh	CS2 + CS3+CS21	1986–1987	Bangladesh	Sequenced	This study
E70	ST-4	LT + STh	CS2 + CS3 +CS21	1986–1987	Bangladesh	Sequenced	This study
E143	ST-4	LT + STh	CS2 + CS3+CS21	1983	Japan	Sequenced	This study
E272	ST-4	LT + STh	CS2 + CS3	1987	Japan	Sequenced	This study
E1624	ST-4	LT + STh	CS2 + CS3+CS21	1994–1995	Indonesia	Sequenced	This study
E1649	ST-4	LT + STh	CS2 + CS3+CS21	1997	Indonesia	Public, LR882973.1	^7^
TW10598	ST-4	LT + STh	CS2 + CS3+CS21	1997	Guinea-Bissau	Public, CP035846.1	^49^
2014EL-1346-6	ST-4	LT + STh	CS2 + CS3+CS21	2014	USA	Public, CP024232.1	^50^
2011EL-1370-2	ST-4	LT + STh	CS2 + CS3+CS21	2011	USA	Public, CP022912.1	^51^
99-3165	ST-4	LT + STh	CS2 + CS3	1999	USA	Public, CP029981.1	^52^
B2C	ST-4	LT + STh	CS2 + CS3+CS21	1971	Viet Nam	Public, CP035874.1	^53^
F5656C1	ST-4	LT + STh	CS2 + CS3	1998	USA	Public, CP024260.1	^50,54^

^*^Complete genome was sequenced in this study or retrieved from GenBank.

**Fig. 2:**
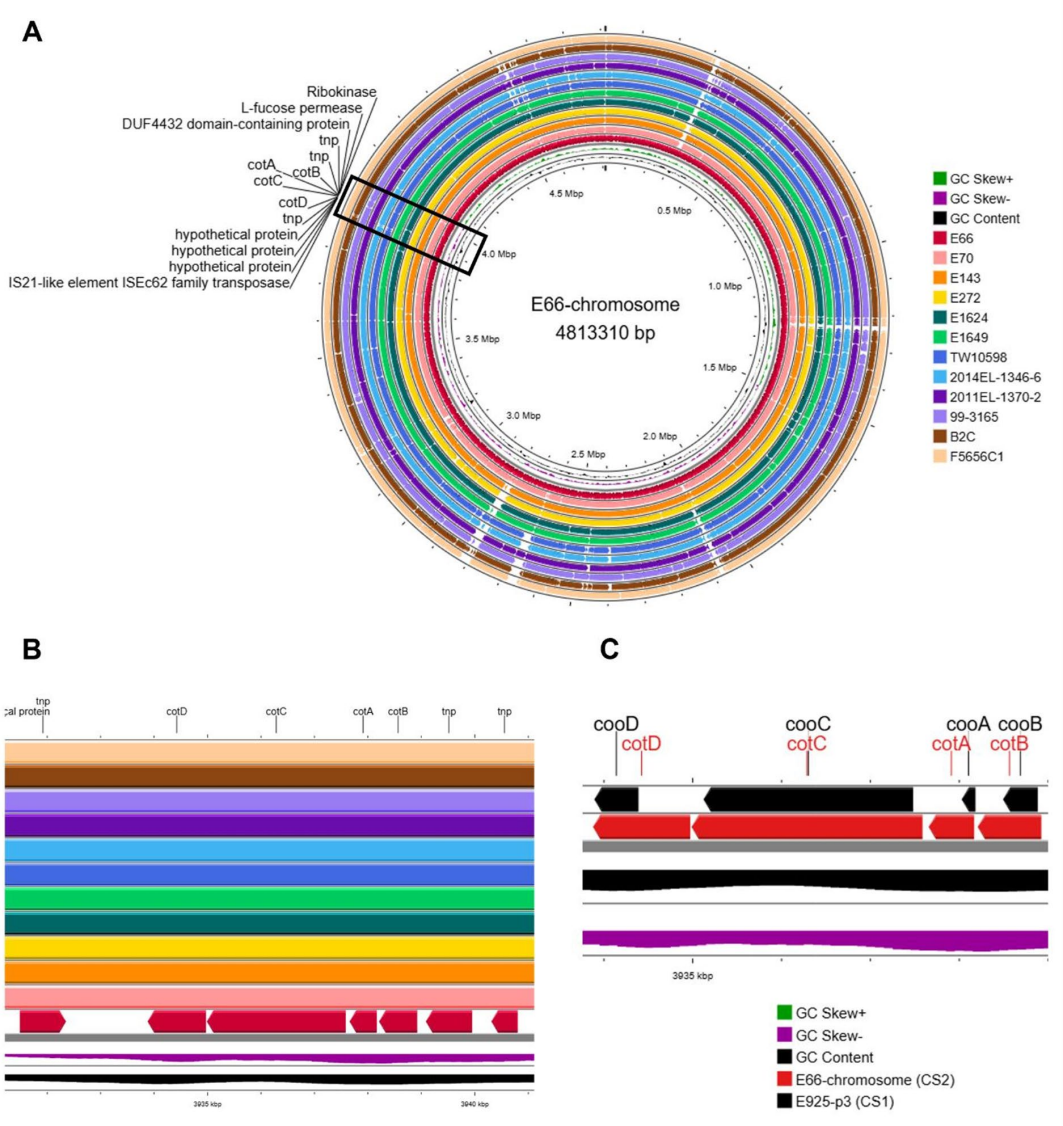
Chromosomal integration of the CS2 colonization factor in ETEC L2 isolates. **(A)** Circular BLASTn alignments of the chromosomes from 12 ETEC L2 isolates, generated with Proksee, using the chromosome of strain E66 as reference. The innermost ring represents the annotated E66 chromosome, and each successive outer ring corresponds to an individual L2 isolate aligned against the reference. The rings display annotated backbone genes (predicted with Bakta), together with GC content and GC skew. **(B)** Zoomed-in view of the CS2 locus (*cotBACD*) and neighbouring genomic regions on the E66 chromosome between 3,930 and 3,940 kb. **(C)** Gene-level comparison between the chromosomal CS2 operon (*cotBACD*) and the plasmid-borne CS1 operon (*cooBACD*).

In contrast to other ETEC lineages, where CFs are exclusively plasmid-encoded, all L2 isolates carried a single CF operon, CS2 (*cotBACD*)^17^, integrated into the chromosome at a conserved locus. This locus was consistently flanked by insertion sequences and transposase fragments across all isolates (Fig. 2A and B). The GC content of the CS2 locus (~ 35%) was substantially lower than the average chromosomal GC content (~ 51%) suggesting horizontal acquisition. Sequence alignment revealed that the chromosomal CS2 operon shares approximately 70% nucleotide identity with the plasmid-borne CS1 (*cooBACD*)^18^ operon found in L1 isolates (Fig. 2C).

Together, these findings indicate that ETEC L2 exhibits a hybrid virulence gene organization, combining chromosomal integration of a key colonization factor with plasmid-mediated accessory virulence traits, in contrast to the fully plasmid-dependent CF architecture observed in other ETEC lineages.

### Fusion of plasmids revealed restructuring of the L2 plasmidome

Complete plasmid sequences were resolved from five fully assembled ETEC L2 genomes generated in this study, while plasmid content and gene composition were assessed across all twelve strains. Each isolate carried between two and five plasmids (p1 to p5), ranging in size from approximately 5 kb to over 120 kb (Table 2).

**Table 2:** Description of the chromosome and plasmids present in the ETEC L2 isolates.

Strain	Genome	Size (bp)	GC content (%)	Inc profile (Alleles)	Virulence factors/ARGs	Accession no*
E66	Chromosome	4,813,310	50.8	-	*cotBACD* (CS2)	JBXYCH010000001.1
	Plasmid p1	118,298	47.0	IncF (FII_11)	*eltAB1* (LT), *estA3/4* (STh), *cstA-G* (CS3), *etpBAC*, *eatA*	JBXYCH010000002.1
	Plasmid p2	74,199	48.4	IncF (FII_6, FIB_45)	*lngX1RSTX2ABCDEFGHIJP* (CS21)	JBXYCH010000003.1
E70	Chromosome	4,853,896	50.8	-	*cotBACD* (CS2)	JBXYCG010000001.1
	Plasmid p1	117,445	47.0	IncF (FII_11)	*eltAB1* (LT), *estA3/4* (STh), *cstA-G* (CS3), *etpBAC*, *eatA*	JBXYCG010000002.1
	Plasmid p2	75,161	48.4	IncF (FII_6, FIB_45)	*lngX1RSTX2ABCDEFGHIJP* (CS21)	JBXYCG010000003.1
	Plasmid p3	5659	43.3	Unassigned	-	JBXYCG010000004.1
	Plasmid p4	5126	49.3	Unassigned	-	JBXYCG010000005.1
E143	Chromosome	4,800,048	50.8	-	*cotBACD* (CS2)	JBXYCF010000001.1
	Plasmid p1	120,152	47.2	IncF (FII_11)	*eltAB1* (LT), *estA3/4* (STh), *cstA-G* (CS3), *etpBAC*, *eatA*	JBXYCF010000002.1
	Plasmid p2	75,169	48.4	IncF (FII_6, FIB_45)	*lngX1RSTX2ABCDEFGHIJP* (CS21)	JBXYCF010000003.1
E272	Chromosome	4,749,640	50.7	-	*cotBACD* (CS2)	JBXYCE010000001.1
	Plasmid p1	117,433	47.0	IncF (FII_11)	*eltAB1* (LT), *estA3/4* (STh), *cstA-G* (CS3), *etpBAC*, *eatA*	JBXYCE010000002.1
	Plasmid p2	102,300	51.5	IncB/O/K/Z	*catA1*, *tetB*, *tetR*	JBXYCE010000003.1
E1624	Chromosome	4,706,392	50.8	-	*cotBACD* (CS2)	JBXYCD010000001.1
	Plasmid p1	120,153	47.2	IncF (FII_11)	*eltAB1* (LT), *estA3/4* (STh), *cstA-G* (CS3), *etpBAC*, *eatA*	JBXYCD010000002.1
	Plasmid p2	75,946	48.4	IncF (FII_6, FIB_45)	*lngX1RSTX2ABCDEFGHIJP* (CS21)	JBXYCD010000003.1
	Plasmid p3	95,407	47.7	IncY	-	JBXYCD010000004.1
E1649	Chromosome	4,721,269	50.8	-	*cotBACD* (CS2)	LR882973.1
	Plasmid p1	120,141	47.2	IncF (FII_11)	*eltAB1* (LT), *estA3/4* (STh), *cstA-G* (CS3), *etpBAC*, *eatA*	LR882976.1
	Plasmid p2	86,517	48.6	IncF (FII_6, FIB_45)	*lngX1RSTX2ABCDEFGHIJP* (CS21)	LR882974.1
	Plasmid p3	102,017	47.6	IncY	-	LR882977.1
	Plasmid p4	8834	42.9	Unassigned	-	LR882975.1
TW10598	Chromosome	4,872,115	50.8	-	*cotBACD* (CS2)	CP035846.1
	Plasmid p1	141,411	48.4	IncF (FII_11)	*eltAB1* (LT), *estA3/4* (STh), *cstA-G* (CS3), *etpBAC*, *eatA*	CP035847.1
	Plasmid p2	61,784	47.0	IncF (FII_111, FIB_45)	*lngX1RSTX2ABCDEFGHIJP* (CS21)	CP035849.1
	Plasmid p3	63,103	50.6	IncF (FII_98), IncFIA	*tetB*, *tetR*	CP035848.1
2014EL-1346-6	Chromosome	4,872,840	50.7	-	*cotBACD* (CS2)	CP024232.1
	Plasmid p1	152,713	48.4	IncF (FII_11)	*eltAB1* (LT), *estA3/4* (STh), *cstA-G* (CS3), *etpBAC*, *eatA*	CP024237.1
	Plasmid p2	62,188	47.1	IncF (FII_111, FIB_45)	*lngX1RSTX2ABCDEFGHIJP* (CS21)	CP024235.1
	Plasmid p3	226,119	46.8	IncF (FIA_8), IncHI	*-*	CP024236.1
	Plasmid p4**	30,162	50.9	IncI1	*-*	CP024233.1
	Plasmid p5**	40,223	49.7	Unassigned	*-*	CP024234.1
2011EL-1370-2	Chromosome	4,785,142	50.7	-	*cotBACD* (CS2)	CP022912.1
	Plasmid p1	117,021	46.9	IncF (FII_11)	*eltAB1* (LT), *estA3/4* (STh), *cstA-G* (CS3), *etpBAC*, *eatA*	CP022914.1
	Plasmid p2	63,392	47.8	IncF (FII_6, FIB_45)	*lngX1RSTX2ABCDEFGHIJP* (CS21)	CP022913.1
99-3165	Chromosome	4,703,465	50.8	-	*cotBACD* (CS2)	CP029981.1
	Plasmid p1	117,740	47.1	IncF (FII_11)	*eltAB1* (LT), *estA3/4* (STh), *cstA-G* (CS3), *etpBAC*, *eatA*	CP029980.1
	Plasmid p2**	36,564	45.0	IncF (FII_59)	*tetB*, *tetR*	CP029979.1
	Plasmid p3	71,543	52.1	IncF (FII_6)	*-*	CP029982.1
B2C	Chromosome	4,829,955	50.8	-	*cotBACD* (CS2)	CP035874.1
	Plasmid p1	151,583	48.4	IncF (FII_11)	*eltAB1* (LT), *estA3/4* (STh), *cstA-G* (CS3), *etpBAC*, *eatA*	CP035875.1
	Plasmid p2	62,180	47.1	IncF (FII_111, FIB_45)	*lngX1RSTX2ABCDEFGHIJP* (CS21)	CP035878.1
	Plasmid p3	83,694	51.8	IncB/O/K/Z	*tetB*, *tetR*	CP035877.1
	Plasmid p4	96,149	47.4	Unassigned	*-*	CP035876.1
F5656C1	Chromosome	4,733,683	50.8	*-*	*cotBACD* (CS2)	CP024260.1
	Plasmid p1	119,846	47.1	IncF (FII_11)	*eltAB1* (LT), *estA3/4* (STh), *cstA-G* (CS3), *etpBAC*, *eatA*	CP024262.1
	Plasmid p2	45,056	40.3	IncX1, IncX3	-	CP024261.1

^*^ New strains: genome assembly accession numbers; other strains: accession numbers from public databases. ^**^Plasmids are linear.

**Fig. 3:**
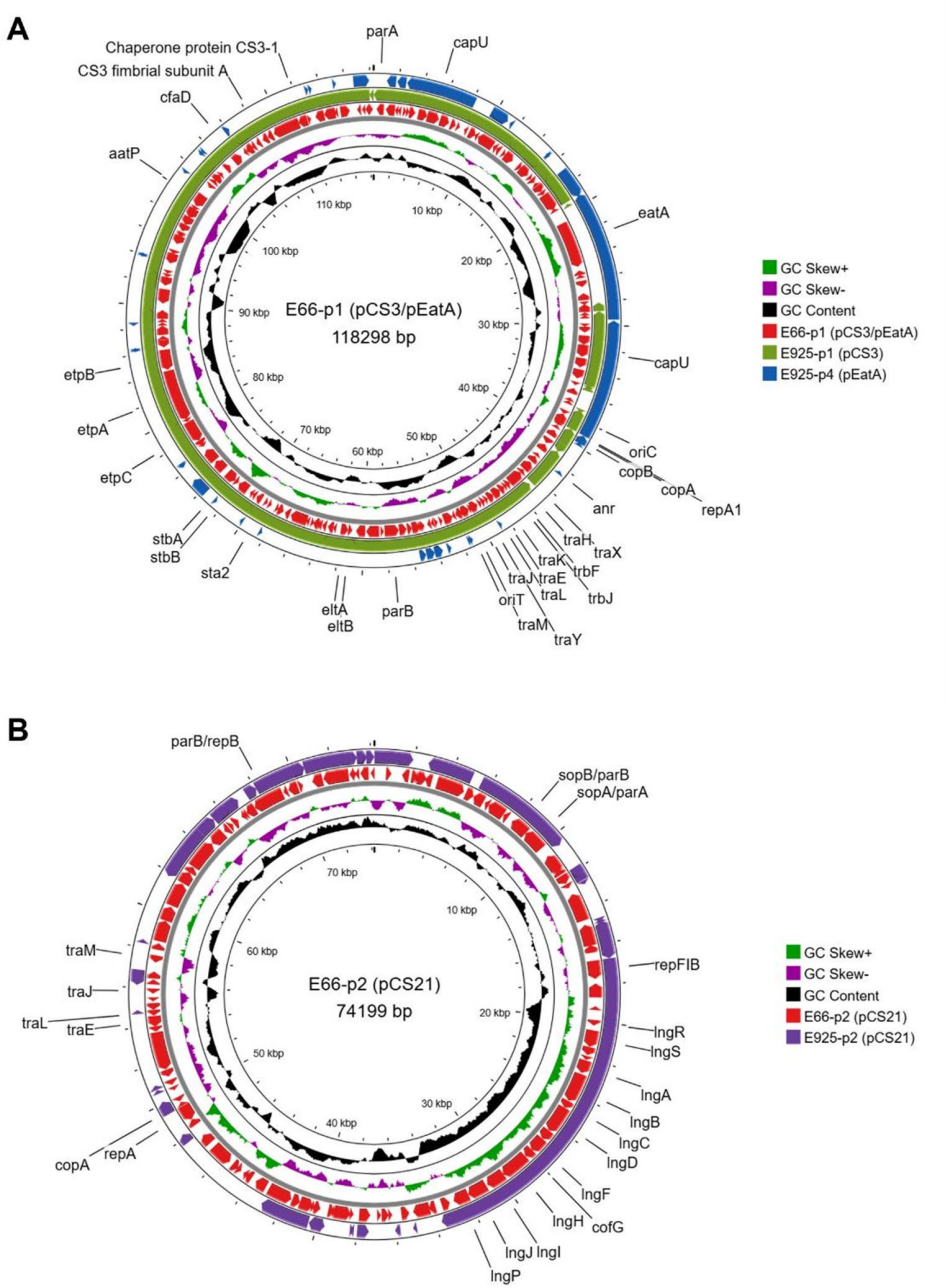
Comparative analysis of the p1 and p2 plasmids in ETEC L2 isolates. **(A)** Circular map of the p1 plasmid (CS3/EatA-encoding) from strain E66, generated with Proksee. The innermost ring represents the annotated E66 p1 plasmid, while outer rings show BLASTn alignments of L1 plasmids pCS3 and pEatA from strain E925 for comparison. The visualization highlights that sequences corresponding to the CS3 and EatA plasmids are combined within a single replicon in L2. **(B)** Circular map of the p2 plasmid (CS21-encoding) from strain E66, generated with Proksee. The innermost ring represents the annotated E66 p2 plasmid, while outer ring shows BLASTn alignments of L1 plasmids pC21 from strain E925 for comparison. Additional rings in both maps display annotated backbone genes (predicted with Bakta), GC content, and GC skew. These maps serve as a representative example of p1(**A**) and p2 (**B**) plasmids observed across the twelve L2 isolates analysed.

Comparison with L1 strains, which consistently harbour four distinct plasmids encoding CS1, CS3, CS21, and EatA (Taheri et al., manuscript under review in *BMC Microbiology*), showed that all L2 isolates contained two large, conserved plasmids, designated p1 (115–120 kb) and p2 (60–80 kb), forming the core L2 plasmidome. These plasmids showed extensive sequence homology to the L1 plasmids harbouring CS3 and CS21. Notably, in L2 isolates, two previously independent plasmids corresponding to L1 CS3 and EatA plasmids are fused into a single plasmid replicon (p1), which retains the core virulence genes (Fig. 3). Comparative analysis using BRIG revealed high conservation in gene content and synteny across isolates from different decades and geographic origins, with plasmid backbones exhibiting ~ 80–100% sequence identity (Fig. 1). Variation was largely restricted to accessory regions. Both p1 and p2 belonged to the *IncF* family, a group of large low-copy-number plasmids with robust partitioning systems that support stable vertical inheritance. Both plasmids carried conjugation-related (*tra*) systems and were therefore predicted to be conjugative.

In addition to the conserved plasmids, eight isolates harboured accessory plasmids lacking canonical ETEC virulence loci but occasionally encoding antibiotic resistance genes or phage-associated sequences (Table 2), thereby contributing to the diversity of the L2 plasmidome.

### Plasmid gene annotation revealed a conserved virulence backbone in L2 plasmidome

To characterize the functional composition of the conserved plasmidome, gene annotation and virulence factor identification were performed using VirulenceFinder, followed by manual curation of plasmid sequences (Table S2).

In all L2 isolates, the largest plasmid p1 carried the *eltIAB-30* operon encoding heat-labile enterotoxin (LTIh-30), *estah-STa3* encoding the human variant of heat-stable enterotoxin STa3, the mucin-degrading serine protease autotransporter gene *eatA*, and the *cstABCDEFGH*^18^ operon encoding CS3 (Fig. 3A). Additional conserved virulence-associated genes included *astA* (heat-stable enterotoxin EAST-1), *capU* (hexosyltransferase homolog), *etpBAC*^19^(non-fimbrial adhesin and TPS transporter), *cfaD* (AraC-like regulator), and *anr* (AraC-family negative regulator). Structural analysis also identified replication regions *IncF (FII_11)*, partitioning genes (*parAB*), recombination-associated loci, and conjugation-related (*tra*) genes consistent with a mosaic plasmid architecture.

The second conserved plasmid, p2, harboured the *lngX1RSTX2ABCDEFGHIJP*^20^^21^ operon encoding the CS21 colonization factor, together with upstream regulatory genes *lngRS* (Fig. 3B). In addition, p2 encoded replication regions *IncF (FIB_45*,* FII_6/FII_111)*, plasmid maintenance genes (*parAB*, and stability-associated loci), and *tra* genes, supporting its role as a stable and transmissible colonization-associated replicon. In three isolates (E272, 99-3165, and F5656C1), p2 encoded CS21 was absent.

### Accessory plasmids carried antibiotic resistance genes and phage-like elements

**Fig. 4:**
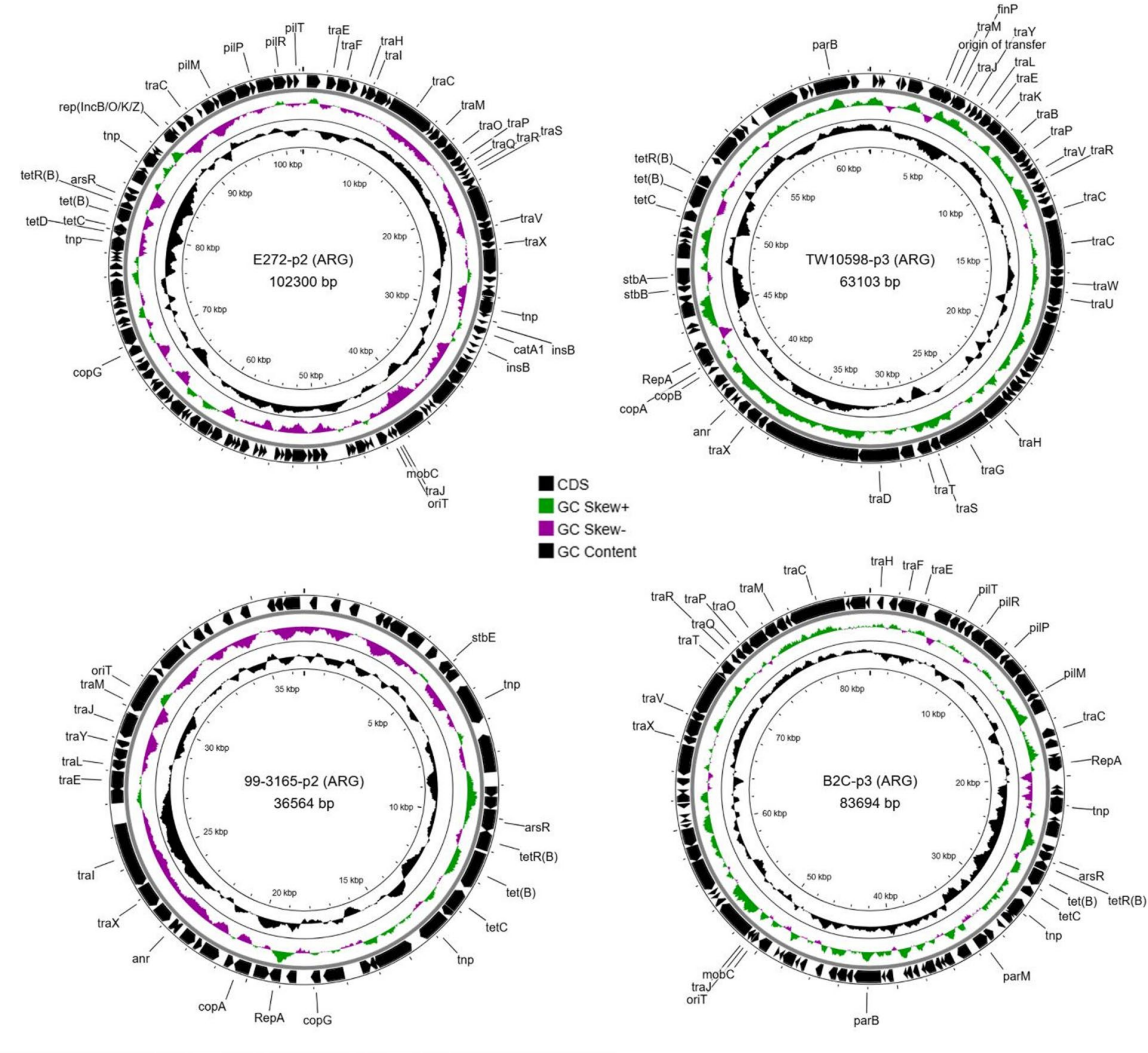
Acquisition of ARG-plasmids in ETEC L2 isolates. Circular maps of four accessory plasmids (ARG-encoding) identified in ETEC L2 isolates, generated with Proksee. Each panel shows a single plasmid, with annotated features, including backbone genes (annotated with Bakta) and ARG-encoding genes. The innermost rings display the GC skew and GC content.

Screening of the accessory plasmids identified antibiotic resistance genes (ARGs) in four of the twelve strains analysed, including E272, TW10598, 99-3165, and B2C (Fig. 4). In all cases, ARGs were plasmid-encoded, and no resistance determinants were detected in the chromosomal sequences. Replicon typing showed that resistance plasmids belonged to different incompatibility groups: plasmid in E272 and B2C were assigned to *IncB/O/K/Z*, while the resistance plasmid in TW10598 and 99-3165 belonged to *IncF*. All four plasmids were predicted to be conjugative, encoding *tra* genes.

Two of the ARG-positive isolates (E727 and 99-3165) lacked the conserved CS21-encoding plasmid p2 and instead carried an alternative plasmid containing resistance genes, whereas TW10598 and B2C retained the CS21 plasmid alongside an additional resistance-associated plasmid (p3). The resistance plasmids carried *tetB* in all four strains and *catA1*in E272, located adjacent to mobile genetic elements such as insertion sequences and transposases, consistent with horizontal acquisition.

**Fig. 5:**
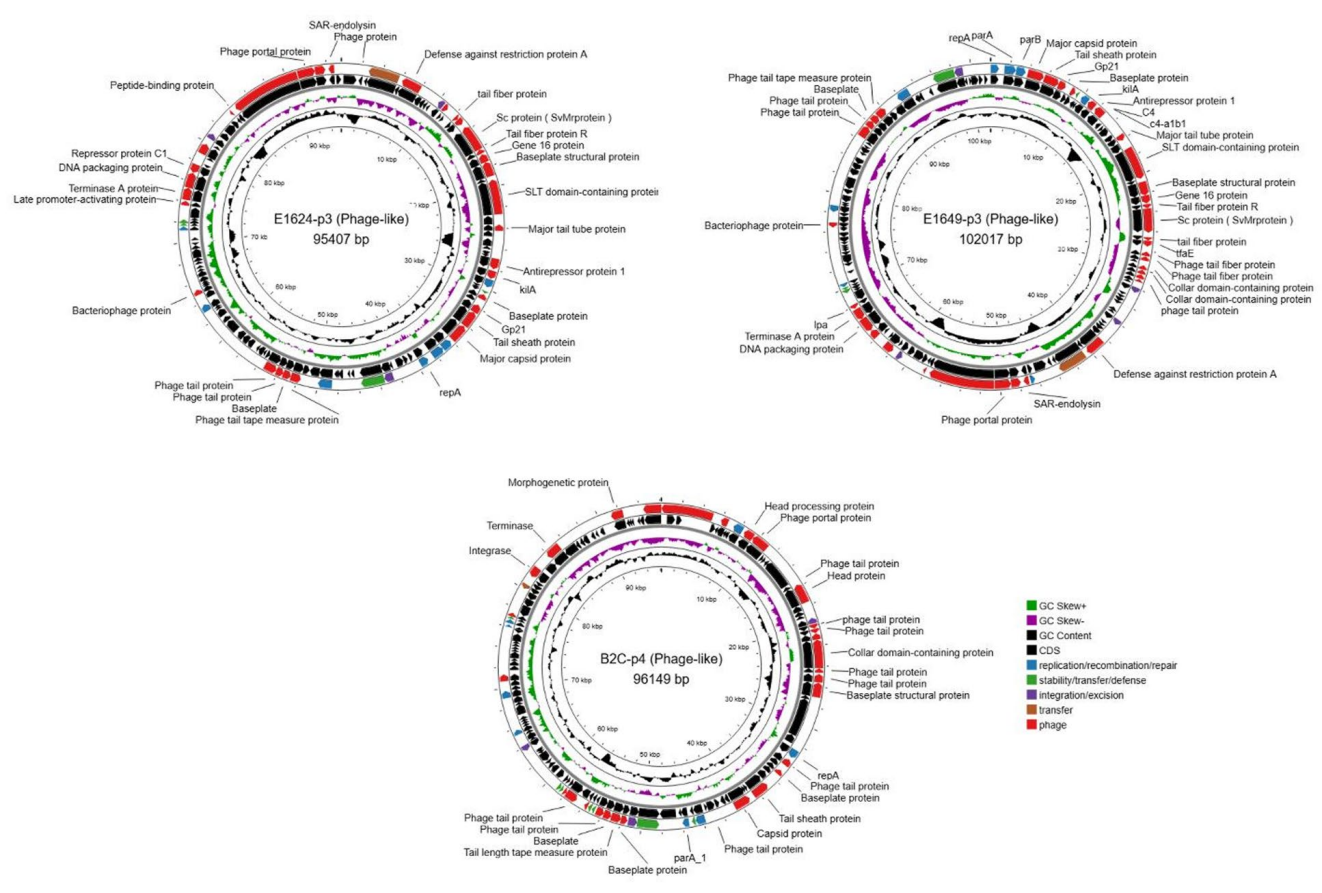
Acquisition of Phage-like plasmids in ETEC L2 isolates. Circular maps of three accessory plasmids (Phage-like) identified in ETEC L2 isolates, generated with Proksee. Each panel shows a single plasmid, with annotated features, including backbone genes (annotated with Bakta) and phage-encoding genes. The innermost rings display the GC skew and GC content.

Phage-like plasmids were identified in three isolates (E1624, E1649, and B2C) (Fig. 5). Replicon analysis showed that the plasmids in E1624 and E1649 belonged to the *IncY* incompatibility group, whereas the plasmid in B2C could not be assigned to a defined incompatibility group. These plasmids encoded clusters of bacteriophage-related genes, including capsid and tail fibre proteins, integrases, and recombinases, consistent with phage-plasmid hybrid elements capable of horizontal transfer. Partial homology was observed with known Enterobacteria phage P1 in plasmids with *IncY* incompatibility group (Figure S2).

## Discussion

Enterotoxigenic *Escherichia coli* (ETEC) isolates cluster into distinct phylogenetic lineages defined by their chromosomal background and plasmid content. Long-read sequencing across the major lineages (L1–L7) has established a robust phylogenetic framework in which lineages are clearly resolved based on core genome structure and lineage-specific virulence plasmids^7,8^.

Lineages 1 and 2 are closely related and share a common ancestral branch, with L1 probably representing a basal lineage and L2 a more recently diverged lineage^8^. Whitin this context, we characterized twelve L2 isolates using complete PacBio assemblies. Although L1 and L2 share a conserved repertoire of canonical virulence determinants, including enterotoxins and colonization factors, these determinants are organized differently at both chromosomal and plasmid levels, reflecting distinct evolutionary strategies.

A defining feature of L2 is the chromosomal integration of CS2, in contrast to L1, where all CFs are plasmid-encoded. Chromosomal localization likely promotes stable vertical inheritance and reduces reliance on plasmid maintenance. The CS2 locus is flanked by insertion sequences and transposase remnants, suggesting acquisition via mobile genetic elements followed by chromosomal fixation. Sequence homology with the L1 plasmid-borne CS1 operon further indicates descent from a shared ancestral operon, followed by lineage-specific divergence and genomic relocation.

Plasmid-to-chromosome transitions of virulence-associated loci have been described in other bacterial pathogens and are thought to represent adaptive responses to selective pressures favouring genetic stability^22,23^. Such transitions may occur when plasmid carriage imposes a fitness cost on the host bacterium^24^. These findings demonstrate that closely related ETEC lineages can preserve equivalent colonization function through distinct genomic configurations.

Despite chromosomal fixation of CS2, L2 retains additional CFs on plasmids, resulting in a hybrid virulence architecture. Long-read sequencing revealed substantial remodelling of the L2 plasmidome. Plasmid p1 results from the fusion of L1 CS3 and EatA plasmids, bringing enterotoxins, CS3, and EatA together within a single plasmid replicon, likely shaped by insertion sequence-mediated recombination^25,26^. EatA facilitates intestinal colonization by degrading mucins and modulating host defences, potentially acting synergistically with CS3^27,28^. The stable co-localization of these virulence determinants may promote coordinated regulation while minimizing the risk of partial loss of virulence functions.

The stable chromosomal integration of CS2, together with the conserved virulence gene content encoded on plasmid p1, suggests that both chromosomal and plasmid-borne virulence determinants are maintained within this lineage. This combined stability indicates that the overall collection of virulence traits is preserved over time and across geographic regions. This may contribute to sustained pathogenic potential and the repeated isolation of closely related clones over several decades and continents.

CS21 is maintained on a second conserved plasmid, p2. Although p2 was occasionally replaced by resistance-associated plasmids, the core virulence plasmids remained highly conserved. This modular configuration contrasts with the fully plasmid-dependent virulence architecture described for L1 (Taheri et al., manuscript under review in *BMC Microbiology*) and highlights the balance between accessory genome plasticity and preservation of a stable virulence core^22,24,29,30^.

In contrast to the conserved chromosomal backbone and core virulence plasmids, ARG-positive and phage-like plasmids were sporadically distributed among L2 isolates, consistent with recent and independent horizontal acquisition events. ARGs were exclusively plasmid-borne, a pattern widely observed in pathogenic *E. coli* and other Gram‑negative pathogens^8,26,29,31^. Plasmids serve as major vectors for clinically relevant resistance determinants across diverse antibiotic classes, and their frequent association with mobile elements such as insertion sequences facilitates ARG mobility and dissemination^32–34^. The identification of homologous resistance plasmids in diverse *E. coli* backgrounds suggests participation in a broader circulating resistance pool that transcends individual lineages^35^.

Phage-like plasmids, including *IncY* elements related to coliphage P1, were identified in a subset of L2 isolates. Although these elements lacked resistance genes in the isolates analysed here, related *IncY* plasmids in other *E. coli* lineages have been shown to carry clinically relevant determinants, including *mcr-1* and extended-spectrum β-lactamases (ESBL), underscoring their potential to mobilize adaptive traits^36,37^. Their sporadic presence highlights the ongoing potential for future acquisition and dissemination of resistance elements within this lineage.

Considering the increasing incidence of CS2 + CS3 positive ETEC cases, often linked with resistance to ESBLs and other antibiotics, L2 may emerge as an important ETEC lineage warranting further investigation of its virulence and resistance determinants. The comparatively low prevalence of resistance genes observed in the present study likely reflects the collection years of the analysed isolates as ESBL and multi-resistance in ETEC has emerged from 2012 and onwards^12^. Additional studies incorporating recently sequenced long‑read L2 genomes will be necessary to capture contemporary accessory genome dynamics.

Finally, this work underscores the importance of long-read sequencing for resolving plasmid fusion events, genomic rearrangements, and accessory genome architecture. Such features are frequently obscured in short-read assemblies, and their accurate resolution provides deeper insight into lineage-specific evolution and virulence organization in this important pathogen.

## Conclusions

This study provides a complete long-read genomic characterization of twelve ETEC L2 isolates and reveals a distinct lineage-specific genome organization shaped by chromosomal integration and plasmid remodelling. Unlike the fully plasmid-dependent virulence architecture of lineage 1, L2 exhibits chromosomal fixation of the CS2 colonization factor alongside conserved plasmids carrying key virulence determinants, including enterotoxins, CS3, CS21, EatA, and EtpBAC. These conserved plasmids maintain core virulence functions, while antibiotic resistance genes and phage-like plasmids occur sporadically within the accessory genome. The conservation of these genomic features across independently sequenced datasets highlights the stability of the L2 genomic architecture. Together, these findings suggest that chromosomal fixation of CS2 combined with streamlined virulence plasmids may represent an evolutionary strategy that enhances genomic stability and may contribute to the epidemiological success of this lineage.

## Methods

### Bacterial strains, genome dataset, and sequence retrieval

Five ETEC L2 isolates were selected for long-read sequencing using the Pacific Biosciences (PacBio) platform. To obtain a more comprehensive understanding of L2 diversity, genome assemblies for the seven publicly available ETEC L2 strains were retrieved from the National Center for Biotechnology Information (NCBI) GenBank database (https://www.ncbi.nlm.nih.gov/). The genomes were downloaded as assembled sequences in FASTA format and used for downstream analyses. To ensure consistency in comparative analyses, only genomes generated using PacBio sequencing technology were included. Detailed isolate information for all strains included in this study is provided in Table 1. For comparative plasmid analyses, reference plasmids from the ETEC L1 strain E925, p1 (GenBank accession: LR883051.1), p2 (GenBank accession: LR883052.1), p3 (GenBank accession: LR883053.1), and p4 (GenBank accession: LR883054.1), were also included^7^.

The strains analysed in this study were obtained from the ETEC strain collection at the University of Gothenburg, Sweden. Ethical approval for their use was granted by the Regional Ethical Review Board in Gothenburg (reference no. 088 − 10). These strains were originally collected from patients with diarrhoea, with informed consent obtained from the patients or their parents/legal guardians. The study was conducted in accordance with the guidelines of the Declaration of Helsinki.

### Bacterial DNA extraction, library preparation, and genome sequencing

Genomic DNA extraction, library preparation, sequencing, and assembly were performed as described previously (Taheri et al., manuscript under review in *BMC Microbiology*). Briefly, ETEC isolates were grown in Luria broth (LB) to an optical density at 600 nm (OD600) of 0.3, and genomic DNA was extracted using the Qiagen Genomic-tip 500/G kit (Qiagen, Hilden, Germany) according to the manufacturer’s instructions. DNA was sheared into ~ 10 kb and ~ 2 kb fragments for library construction using the GeneMachines HydroShear (Digilab, Marlborough, MA, USA) and Covaris instruments (Covaris, Woburn, MA, USA), respectively. SMRTbell libraries were prepared and sequenced on the PacBio RSII platform (Pacific Biosciences, Menlo Park, CA, USA).

Long-read assemblies were generated using the Hierarchical Genome Assembly Process (HGAP3) pipeline from SMRT portal v2.3.0 (https://github.com/PacificBiosciences/SMRT-Analysis/) with default settings for 10-kb libraries, while the 2-kb libraries were assembled using the Falcon pipeline (Pacific Biosciences, Menlo Park, CA, USA). Circlator v1.1.0 (https://sanger-pathogens.github.io/circlator/) was used to remove small self-contained contigs and circularize remaining sequences. Error correction was performed with Quiver by aligning corrected reads back to the circularized genome. Genome annotation was conducted with Prokka v1.14.6^38^, using the ETEC reference proteome E24377A (GenBank accession: CP000800.1) as the primary annotation source. Assembly statistics were compiled using MultiQC v1.0 (https://seqera.io/multiqc/)^39^.

### Functional annotation, plasmid characterization, and comparative genomics

Functional analyses were performed primarily using the Centre for Genomic Epidemiology (CGE) pipeline (https://www.genomicepidemiology.org/). Antibiotic resistance genes (ARGs) were identified using ResFinder v4.7.2^40^, while virulence genes, multilocus sequence types, and serotypes were determined using VirulenceFinder v2.0.5^41^, MLST v2.0.9^42^, and SerotypeFinder v2.0.1^43^, respectively. Plasmid incompatibility groups were assigned using PlasmidFinder v2.0.2 and pMLST v0.1.0^44^.

Plasmid annotation, comparison, and visualization were performed using Proksee (https://proksee.ca/projects)^45^, incorporating functional annotation generated by Bakta and the mobile genetic element database MobileOG-db^46,47^. Genome size and GC content were also extracted as part of the annotation workflow.

Comparative analyses of chromosomal and plasmid sequences were performed using BLASTn, and synteny was visualized using Blast Ring Image Generator (BRIG) v0.95^48^ to assess genomic similarity among the twelve isolates.

## Supplementary Information

Below is the link to the electronic supplementary material.

Supplementary Material 1

## Data Availability

The whole genome sequence data generated in this study have been deposited in the NCBI GenBank database under BioProject accession number PRJNA1424559 (https://www.ncbi.nlm.nih.gov/bioproject/1424559). The whole genome shotgun projects have been deposited in DDBJ/ENA/GenBank under the following accession numbers: JBXYCH010000000 (https://www.ncbi.nlm.nih.gov/nuccore/JBXYCH000000000.1), JBXYCG010000000 (https://www.ncbi.nlm.nih.gov/nuccore/JBXYCG000000000.1), JBXYCF010000000 (https://www.ncbi.nlm.nih.gov/nuccore/JBXYCF000000000.1), JBXYCE010000000 (https://www.ncbi.nlm.nih.gov/nuccore/JBXYCE000000000.1), and JBXYCD010000000 (https://www.ncbi.nlm.nih.gov/nuccore/JBXYCD000000000.1). The version described in this study is version 1.
