# Supplementary material for: Genomic characterization of Enterotoxigenic Escherichia coli lineage 2 (CS2 + CS3) by long-read sequencing reveals distinct lineage-specific genome organization
Source: Sci Rep. 2026 May 26;16:16289. doi: 10.1038/s41598-026-55068-w (PMC13212926; doi:10.1038/s41598-026-55068-w)
Supplement: Supplementary file 1 — Supplementary Material 1 [file 41598_2026_55068_MOESM1_ESM.pdf]

Genomic characterization of Enterotoxigenic *Escherichia coli* lineage 2 (CS2+CS3) by long-read sequencing reveals distinct lineage-specific genome organization.

Nayyer Taheri[1,*], Åsa Sjöling[1,2]

[1]Department of Chemistry and Molecular Biology, University of Gothenburg, Gothenburg, Sweden.

[2]Department of Microbiology, Tumor and Cell Biology, Karolinska Institute, Stockholm, Sweden.

[*]Correspondence to Nayyer Taheri, Email: nayyer.taheri@gu.se

Figure S1

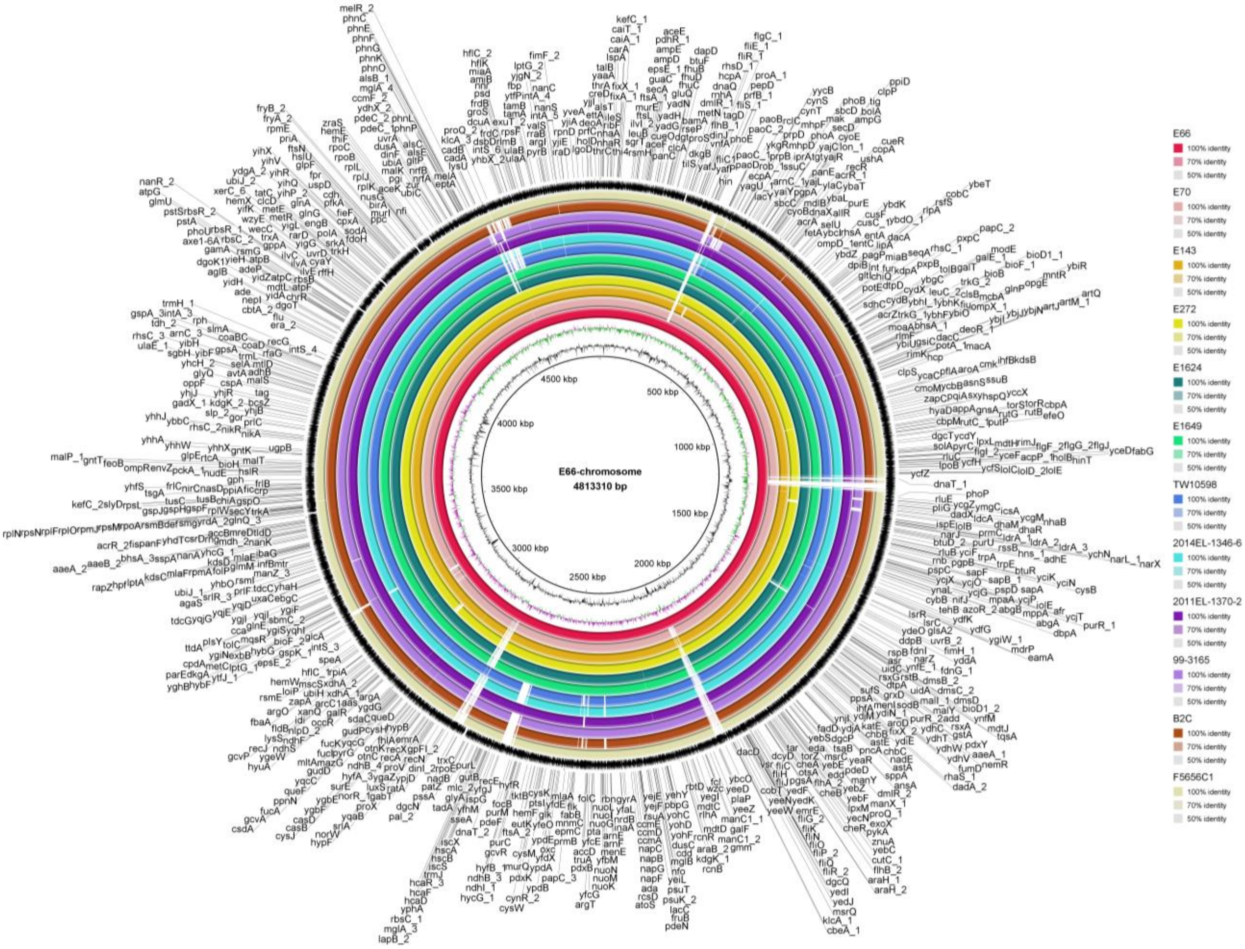

Figure S1. Comparative analysis of chromosome in ETEC L2 isolates.

Circular BLASTn alignment of chromosomes from twelve ETEC L2 isolates, using the chromosome of strain E66 as reference, generated with BRIG. The outermost ring represents the annotated E66 chromosome, including labelled backbone genes, while each concentric inner ring corresponds to a chromosome from one of the other L2 isolates aligned against the reference. Ring colour intensity reflects nucleotide identity to E66, with darker shading indicating higher similarity and lighter shading lower similarity; white regions indicate sequences absent in E66. The innermost rings display GC content and GC skew of the E66 chromosome.

Figure S2

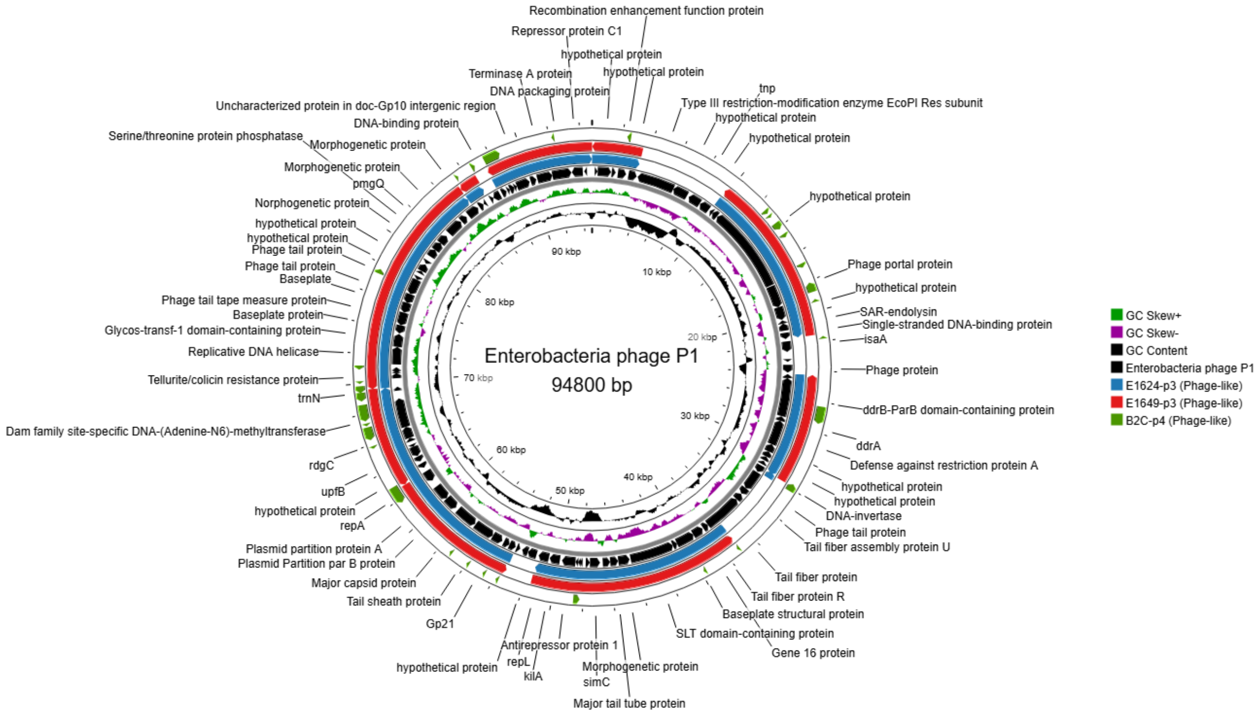

Figure S2. Comparative analysis of Phage-like plasmids in ETEC L2 isolates.

Circular BLASTn alignment of Phage-like plasmids from three ETEC L2 isolates using Enterobacteria phage P1 as a reference, generated with Proksee. The innermost ring represents the annotated P1 genome (predicted with Bakta), while the outer rings show BLASTn alignments of the Phage-like plasmids. Additional rings display GC content and GC skew of the P1 genome.

Table S1. Genomic features of the chromosome in the ETEC L2 isolates.

Strains	Size (bp)	Contig	GC content (%)	Predicted CDS	Signal peptides	tRNAs	rRNAs	tmRNA	Plasmid no.
E66	4813310	1	50.8	4585	428	88	22	1	2
E70	4853896	1	50.8	4605	432	88	22	1	4
E143	4800048	1	50.8	4568	431	88	22	1	2
E272	4749640	1	50.7	4495	428	87	22	1	2
E1624	4706392	1	50.8	4423	422	88	22	1	3

Table S2. Virulence genes predicted by VirulenceFinder in the ETEC L2 isolates.

Genome	Virulence gene	Protein function	E66	E70	E143	E272	E1624	E1649	TW10598	2014 EL	2011 EL	99-3165	B2C	F5656C1
chromosome	cotA	CS2 Major pilin chaperone	+	+	+	+	+	+	+	+	+	+	+	+
	cotB	CS2 Major pilin chaperone	+	+	+	+	+	+	+	+	+	+	+	+
	cotC	CS2 Usher	+	+	+	+	+	+	+	+	+	+	+	+
	cotD	CS2 Chaperone	+	+	+	+	+	+	+	+	+	+	+	+
	csgA	curlin major subunit CsgA	+	+	+	+	+	+	+	+	+	+	+	+
	fimH	Type 1 fimbriae	+	+	+	+	+	+	+	+	+	+	+	+
	gad	Glutamate decarboxylase	+	+	+	+	+	+	+	+	+	+	+	+
	hlyE	Avian *E. coli* haemolysin	+	+	+	+	+	+	+	+	-----	+	+	+
	iss	Increased serum survival	+	+	+	+	+	+	+	+	+	+	+	+
	kpsE	Capsule polysaccharide export inner-membrane protein	+	+	+	+	+	+	+	+	+	+	+	+
	kpsM_K15	Polysialic acid transport protein; Group 3 capsule	+	+	+	+	+	+	+	+	+	+	+	+
	nlpI	lipoprotein NlpI precursor	+	+	+	+	+	+	+	+	+	+	+	+
	ompT	Outer membrane protease (protein protease 7)	+	+	+	+	+	+	+	+	+	+	+	+
	terC	Tellurium ion resistance protein	+	+	+	+	+	+	+	+	+	+	+	+
	tia	Tia Invasion determinant	+	+	+	+	+	+	-----	-----	+	+	-----	+
	yehA	Outer membrane lipoprotein, YHD fimbriael cluster	+	+	+	+	+	+	+	+	+	+	-----	+
	yehB	Usher, YHD fimbriael cluster	+	+	+	-----	+	+	+	+	-----	+	+	+
	yehD	Major pilin subunit, YHD fimbriael cluster	+	+	+	+	+	+	+	+	+	+	+	+
p1 (pCS3/pEatA)	anr	AraC negative regulator	+	+	+	+	+	+	+	+	+	+	+	+
	astA	Heat-stable enterotoxin EAST-1	+	+	+	+	+	+	-----	+	+	+	+	+
	capU	Hexosyltransferase homolog	+	+	+	+	+	+	+	+	+	+	+	+
	cfaD	CFA/1 Minor pilin	+	+	+	+	+	+	+	+	+	+	+	+
	cstA	CS3 Chaperone	+	+	+	+	+	+	+	+	+	+	+	+
	cstB	CS3 Usher	-----	-----	-----	-----	-----	-----	+	-----	-----	-----	-----	-----
	cstE	CS3	-----	-----	-----	-----	+	-----	-----	-----	-----	-----	-----	-----
	cstF	CS3	+	+	+	+	-----	+	-----	+	+	+	+	+
	cstG	CS3 Major pilin	+	+	+	+	+	+	+	+	+	+	+	+
	eatA	Mucin-degrading serine protease	+	+	+	+	+	+	+	+	-----	+	+	+

	eltIAB-30	Heat-labile enterotoxin LTIh-30	+	+	+	+	+	+	+	+	+	+	+	+
	estah-STa3	Heat-stable enterotoxin STa3 human variant	+	+	+	+	+	+	+	+	+	+	+	+
	etpA	EtpA Invasin and adherence	+	+	+	+	+	+	-----	-----	+	+	-----	-----
	etpB	EtpB Nonfimbrial adhesin/TPS transporter	+	+	+	+	+	+	+	+	+	+	+	+
	etpC	EtpC Glycotransferase	-----	+	-----	-----	+	+	+	+	-----	-----	+	+
	traT	Outer membrane protein complement resistance	-----	-----	-----	-----	-----	-----	+	+	-----	-----	+	-----
p2 (pCS21)	anr	AraC negative regulator	-----	-----	-----	-----	-----	-----	+	+	-----	-----	+	-----
	lngA	CS21 Major pilin	-----	-----	-----	-----	-----	-----	+	+	-----	-----	+	-----
	lngB	CS21	+	+	+	-----	+	+	+	+	+	-----	+	-----
	lngC	CS21	+	+	+	-----	+	+	+	+	+	-----	+	-----
	lngD	CS21 Putative outer membrane protein	+	+	+	-----	+	+	-----	+	+	-----	+	-----
	lngE	CS21 Putative inner membrane protein	+	+	+	-----	+	+	+	+	+	-----	+	-----
	lngG	CS21 Putative periplasmic protein	+	+	+	-----	+	+	+	+	+	-----	+	-----
	lngH	CS21 Putative nucleotide-binding protein	+	+	+	-----	+	+	+	+	+	-----	+	-----
	lngI	CS21 Putative inner membrane protein	+	-----	+	-----	+	+	+	+	-----	-----	+	-----
	lngJ	CS21 Putative ATPase-like	+	+	+	-----	+	+	+	+	+	-----	+	-----
	lngP	CS21 Putative prepilin peptidase	+	+	+	-----	+	+	+	+	+	-----	+	-----
	lngR	CS21 Putative papB-like regulator	+	+	+	-----	+	+	+	+	+	-----	+	-----
	lngS	CS21 Putative AraC-like regulator	+	+	+	-----	+	+	+	+	+	-----	+	-----
	lngT	CS21	+	+	+	-----	+	+	+	+	+	-----	+	-----
	lngX1	CS21	-----	-----	-----	-----	-----	-----	+	+	-----	-----	+	-----
	lngX2	CS21	+	+	+	-----	+	+	+	+	+	-----	+	-----
Accessory plasmids	traT	Outer membrane protein complement resistance	-----	-----	-----	+	-----	-----	+	-----	-----	-----	+	-----
	traJ	Protein TraJ (Positive regulator of conjugal transfer operon)	-----	-----	-----	-----	-----	-----	-----	-----	-----	+	-----	-----
	hlyA	Hemolysin A	-----	-----	-----	-----	-----	-----	-----	+	-----	-----	-----	-----
